# Risk factors for distal migration of biliary plastic stents and related duodenal injury

**DOI:** 10.1007/s00464-019-06957-x

**Published:** 2019-07-18

**Authors:** Xiang-lei Yuan, Lian-song Ye, Qin Liu, Chun-cheng Wu, Wei Liu, Xian-hui Zeng, Yu-hang Zhang, Lin-jie Guo, Yu-yan Zhang, Yan Li, Xin-yue Zhou, Bing Hu

**Affiliations:** Department of Gastroenterology, West China Hospital, Sichuan University, No. 37 Guo Xue Xiang, Chengdu, 610041 Sichuan China

**Keywords:** Endoscopic biliary stenting, Distal migration, Duodenal injury

## Abstract

**Background:**

The risk factors of duodenal injury from distal migrated biliary plastic stents remain uncertain. The aim of this study was to determine the risk factors of distal migration and its related duodenal injury in patients who underwent placement of a single biliary plastic stent for biliary strictures.

**Methods:**

We retrospectively reviewed all patients with biliary strictures who underwent endoscopic placement of a single biliary plastic stent from January 2006 to October 2017.

**Results:**

Two hundred forty-eight patients with 402 endoscopic retrograde cholangiopancreatography procedures were included. The incidence of distal migration was 6.2%. The frequency of duodenal injury was 2.2% in all cases and 36% in cases with distal migration. Benign biliary strictures (BBS), length of the stent above the proximal end of the stricture (> 2 cm), and duration of stent retention (< 3 months) were independently associated with distal migration (*p *= 0.018, *p *= 0.009, and *p *= 0.016, respectively). Duodenal injury occurred more commonly in cases with larger angle (≥ 30°) between the distal end of the stent and the centerline of the patient’s body (*p *= 0.018) or in cases with stent retention < 3 months (*p *= 0.031).

**Conclusions:**

The risk factors of distal migration are BBS and the length of the stent above the proximal end of the stricture. The risk factor of duodenal injury due to distal migration is large angle (≥ 30°) between the distal end of the stent and the centerline of the patient’s body. Distal migration and related duodenal injury are more likely to present during the early period after biliary stenting.

Endoscopic placement of biliary plastic stents for biliary disease was initially described in 1980 by Soehendra and Reynders-Frederix [1]. Subsequently, endoscopic stenting has become a well-established procedure for either benign or malignant strictures that are not suitable for surgery [2–4]. Although biliary stenting is safe in most cases, complications such as stent migration and its related bowel injury need urgent treatment or may even be fatal [5–9].

Generally, the most common site of bowel injury due to stent migration is the duodenum, and distal migration is the main cause [6–8, 10, 11]. Distal migration of biliary plastic stents occurs in up to 6% of cases [11–13]. Although less than 1% of migrated plastic stents will cause duodenal perforation [14, 15], immediate endoscopic removal of the stent is usually needed once it occurs, and further surgical interventions may be indicated [7, 10, 16]. Considering the risks and costs, it is important to determine and avoid the risk factors of stent distal migration and its related duodenal injury. However, to our knowledge, only limited case reports reported possible risk factors of duodenal injury from distal migrated biliary plastic stents [6, 17].

In the present study, we aimed to determine the frequency and risk factors of distal migration and its related duodenal injury in patients who underwent placement of a single biliary plastic stent.

## Methods

### Patients

We retrospectively reviewed the medical records of all patients with biliary strictures who underwent endoscopic placement of a single biliary plastic stent at West China Hospital, Sichuan University, from January 2006 to October 2017. Each procedure was regarded as a separate event. During the study period, a total of 251 patients with 405 endoscopic retrograde cholangiopancreatography (ERCP) procedures were identified, but 3 procedures in 3 patients without available outcome data were excluded. Thus, 248 patients with 402 procedures were included. The study was approved by West China Hospital of Sichuan University Biomedical Research Ethics Committee. Informed consent was obtained from all patients before the initiation of the study.

### Data extraction

**Fig. 1:**
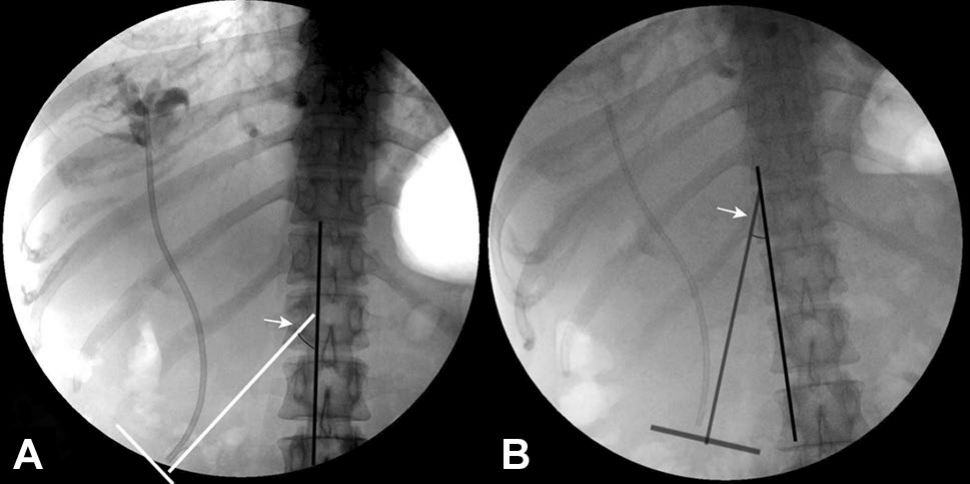
Fluoroscopy demonstrated two stent placements in the same patient. **A** Duodenal injury occurred when the angle between the distal end of the stent and the centerline of the patient’s body was large (41°). **B** No injury was noted when the angle was small (20°)

Patient characteristics and stent details were collected and analyzed by two physicians, X.L.Y, and Q.L, respectively. Baseline patient characteristics included age, sex, clinical diagnosis or indication for stent placement, and location of biliary stricture. Stent details included stent diameter, stent length, length of the stent above the proximal end of the stricture (assessed by fluoroscopy), length of the stent outside of the duodenal papilla (assessed by both endoscopy and fluoroscopy), direction of stent migration (distal or proximal), and duration of stent retention. We also measured the angle between the distal end of the stent and the centerline of the patient’s body on fluoroscopic images at time of stent placement in cases with distal migration (using AutoCAD 2014, with the resulting values rounded up to the nearest whole number) (Fig. 1).

### Procedures

All ERCPs were performed by one of four experienced endoscopists (≥ 300 ERCPs per year). Patients were placed in the prone position and under either conscious sedation or anesthesia. A standard duodenoscope (JF-240, JF-260 V, TJF-240, or TJF-260 V; Olympus Medical Systems, Tokyo, Japan) was used. The endoscopist determined if sphincterotomy was necessary. Brush cytology was performed in patients with unclear diagnosis before stent placement. Under the guidance of a guide wire (Jagwire; Boston Scientific, Natick, MA. United States), a straight plastic stent (Cotton-Leung Biliary stent; Cook Ireland Ltd, Limerick, Ireland) was advanced into the bile duct above the proximal end of the stricture, leaving the distal end outside of the papilla. The diameter of the stents used were 7–12 Fr with a length of 5–15 cm. Procedure success was defined as the placement of the stent across the stricture, as verified by fluoroscopy. Follow-up ERCP was scheduled in 3–6 months for routine stent exchange or removal.

### Definitions

The location of the stricture was divided into two types: proximal (stricture located in the common hepatic duct) and distal (stricture located in the common bile duct) [13]. Distal migration was defined if the location of the stent was below the original position or the stent was not present on endoscopic and fluoroscopic re-examination. Proximal migration was defined if the stent was seen in the bile duct on fluoroscopic re-examination and the distal end of the stent was not observed at the major papilla on endoscopy [13]. Duodenal injury was confirmed if the migrated stent was stuck in the duodenum, resulting in erosion or perforation.

### Statistical analysis

Continuous variables were expressed as mean ± standard deviation. Categorical variables were expressed as frequency or proportion. Chi-square test, Fisher exact test, and logistic regression analysis were performed whenever appropriate. Statistical analysis was performed using SPSS 23.0 (IBM Corp, Armonk, NY). A *p* value < 0.05 was defined as statistically significant.

## Results

### Patient characteristics

In the present study, 248 patients (male, 159; female, 89; mean age, 57.6 ± 18.3 years) with 402 ERCPs were included in the final analysis. Among them, 157 (39.1%) and 245 (60.9%) ERCPs were performed in 106 (42.7%) patients with benign biliary strictures (BBS) and 142 (57.3%) patients with malignant biliary strictures (MBS), respectively (Table 1).

**Table 1:** Patient characteristics

Characteristics	Value
Age (years)	57.6 ± 18.3
Sex (male/female)	159/89
ERCPs	402
Benign biliary stricture	157 (39.1)
Post-liver transplantation	80 (51.0)
Post-cholecystectomy	37 (23.6)
Chronic pancreatitis	25 (15.9)
Portal hypertensive biliopathy	8 (5.1)
Hydatid disease	6 (3.8)
Primary sclerosing cholangitis	1 (0.6)
Malignant biliary stricture	245 (60.9)
Hilar cholangiocarcinoma	98 (40.0)
Distal cholangiocarcinoma	58 (23.7)
Pancreatic cancer	49 (20.0)
Oddi tumor	25 (10.2)
Metastatic cancer	8 (3.3)
Gall balder cancer	7 (2.8)

Values are presented as mean ± standard deviation or n (%)

*ERCP* endoscopic retrograde cholangiopancreatography

### Stent migration and related duodenal injury

Among the 402 cases, 25 distal migrations were noted in 22 patients (once in 19 and twice in 3). The incidence of distal migration was 6.2% (25/402). Duodenal injury from distal migrated stent was noted in 8 patients and occurred 9 times in total, with a frequency of 2.2% (9/402) in all cases and 36% (9/25) in cases with distal migration. Duodenal perforation was noted in 1 case (0.2%, 1/402), which was successfully closed by endoclips. All distal migrated stents were retrieved endoscopically. Details of cases with distal migration-induced duodenal injury are shown in Table 2.

**Table 2:** Characteristics of cases with distal migration-induced duodenal injury

Case/patient	Sex/age (years)	Diagnosis	Stent diameter (Fr)/length (cm)	Length of the stent above the proximal end of the stricture (cm)	length of the stent outside of the duodenal papilla (cm)	Angle (°)^a^	Duration of stent retention	Endoscopic findings in the duodenum^b^
1/1	F/41	Portal hypertensive biliopathy	8.5/15	3	1	41	9 months	Erosion
2/2	M/38	Post-cholecystectomy	10/9	2	1	34	11 months	Erosion
3/3	M/63	Metastatic cancer	8.5/15	1	1	49	1.5 months	Erosion
4/4	F/39	Portal hypertensive biliopathy	8.5/15	2	1	38	2 months	Erosion
5/5	F/74	Pancreatic cancer	8.5/7	3	1	26	3 days	Erosion
6/5	F/74	Pancreatic cancer	10/8	3	1	35	3 days	Perforation
7/6	M/9	Post-liver transplantation	8.5/12	2	1	43	6 months	Erosion
8/7	M/69	Hilar cholangiocarcinoma	8.5/12	2	1	41	2.5 months	Erosion
9/8	M/53	Post-liver transplantation	8.5/12	2	1.5	65	2 months	Erosion

*M* male, F female

^a^The angle between the distal end of the stent and the centerline of the patient’s body was measured by AutoCAD 2014, with the resulting values rounded up to the nearest whole number

^b^The migrated stent was stuck in the duodenum, resulting in erosion or perforation

Twenty proximal migrations (5.0%, 20/402) were noted in 14 patients (once in 10, twice in 3, and four times in 1). There was no proximal migration-related duodenal injury; thus, further analysis was not performed.

### Risk factors of distal migration

The comparison of cases with and without distal migration is shown in Table 3. When compared with MBS, the frequency of distal migration was significantly higher in BBS (10.2% vs. 3.7%, *p *= 0.008). Distal migration occurred more frequently in cases with proximal stricture (12.6% vs. 3.8%, *p *= 0.001) or longer stent (≥ 10 cm) (10.6% vs. 2.7%, *p *= 0.001). We also found that the length of the stent above the proximal end of the stricture (> 2 cm) was associated with higher frequency of distal migration (23.5% vs. 5.5%, *p *= 0.016). However, according to multivariable logistic regression analysis, only BBS, length of the stent above the proximal end of the stricture (> 2 cm), and duration of stent retention (< 3 months) were significantly associated with distal migration (*p *= 0.018, *p *= 0.009, and *p *= 0.016, respectively) (Table 4).

**Table 3:** Risk factors of distal migration based on univariate analysis

	Distal migration (*n *= 25)	Non-distal migration (*n *= 377)	*p*
Etiologies			0.008^a^
Benign biliary stricture (*n *= 157)	16 (10.2)	141 (89.8)	
Malignant biliary stricture (*n *= 245)	9 (3.7)	236 (96.3)	
Stricture location			0.001^a^
Proximal (*n *= 111)	14 (12.6)	97 (87.4)	
Distal (*n *= 291)	11 (3.8)	280 (96.2)	
Stent diameter			0.055^b^
7 Fr (*n *= 13)	1 (92.3)	12 (7.7)	
8.5 Fr (*n *= 194)	18 (9.3)	176 (90.7)	
10 Fr (*n *= 193)	6 (3.1)	187 (96.9)	
12 Fr (*n *= 2)	0	2 (100)	
Stent length			0.001^a^
≥ 10 cm (*n *= 180)	19 (10.6)	161 (89.4)	
< 10 cm (*n *= 222)	6 (2.7)	216 (97.3)	
Length of the stent above the proximal end of the stricture			0.016^b^
> 2 cm (*n *= 17)	4 (23.5)	13 (76.5)	
≤ 2 cm (*n *= 385)	21 (5.5)	364 (94.5)	
Length of the stent outside of the duodenal papilla			0.492^b^
> 1 cm (*n *= 37)	3 (8.1)	34 (91.9)	
≤ 1 cm (*n *= 365)	22 (6.0)	343 (94.0)	
Duration of stent retention			0.050^a^
< 3 months (*n *= 83)	9 (10.8)	74(89.2)	
≥ 3 months (*n *= 319)	16 (5.0)	303 (95.0)	

Values are presented as *n* (%)

^a^Chi-square test

^b^Fisher exact test

**Table 4:** Risk factors of distal migration based on multivariate analysis

	*p*^a^	Exp(B)	95% CI
BBS	0.018	2.966	1.209-7.276
Proximal stricture	0.161	2.239	0.725-6.918
Stent length (≥ 10 cm)	0.143	2.619	0.723-9.492
Length of the stent above the proximal end of the stricture (> 2 cm)	0.009	5.668	1.547-20.767
Duration of stent retention (< 3 months)	0.016	3.107	1.236-7.807

Exp(B): odds ratio; CI: confidence interval; BBS: benign biliary strictures

^a^Logistic regression

### Risk factors of distal migration-induced duodenal injury

The comparison of distal migrated cases with and without duodenal injury is shown in Table 5. Although the incidence of duodenal injury was higher in cases with > 2 cm of stent length above the proximal end of the stricture (75% vs. 28.6%), no significant difference was noted (*p *= 0.116). Duodenal injury occurred more commonly in cases with larger angle (≥ 30°) between the distal end of the stent and the centerline of the patient’s body (57.1% vs. 9.1%, *p *= 0.018). Furthermore, when compared with stent retention ≥ 3 months, the frequency of injury was significantly higher in cases with < 3 months (66.7% vs. 18.8%, *p *= 0.031).

**Table 5:** Risk factors of distal migration-induced duodenal injury based on univariate analysis

	Injury (*n *= 9)	Non-injury (*n *= 16)	*p*
Etiologies			0.671^a^
Benign biliary stricture (*n *= *n *= 16)	5 (31.3)	11 (68.8)	
Malignant biliary stricture (*n *= 9)	4 (44.4)	5 (55.6)	
Stricture location			1.000^a^
Proximal (*n *= 14)	5 (35.7)	9 (64.3)	
Distal (*n *= 11)	4 (36.4)	7 (63.6)	
Stent diameter			1.000^a^
7 Fr (*n *= 1)	–	1 (100)	
8.5 Fr (*n *= 18)	7 (38.9)	11 (61.1)	
10 Fr (*n *= 6)	2 (33.3)	4 (66.7)	
Stent length			0.630^a^
≥ 10 cm (*n *= 19)	6 (31.6)	13 (68.4)	
< 10 cm (*n *= 6)	3 (50.0)	3 (50.0)	
Length of the stent above the proximal end of the stricture			0.116^a^
> 2 cm (*n *= 4)	3 (75.0)	1 (25.0)	
≤ 2 cm (*n *= 21)	6 (28.6)	15 (71.4)	
Length of the stent outside of the duodenal papilla			1.000^a^
> 1 cm (*n *= 3)	1 (33.3)	2 (66.7)	
≤ 1 cm (*n *= 22)	8 (36.4)	14 (63.6)	
Duration of stent retention			0.031^a^
< 3 months (*n *= 9)	6 (66.7)	3 (33.3)	
≥ 3 months (*n *= 16)	3 (18.8)	13 (81.2)	
Angle^b^			0.018^a^
≥ 30° (*n *= 14)	8 (57.1)	6 (42.9)	
< 30° (n = 11)	1 (9.1)	10 (90.9)	

^a^Fisher exact test

^b^The angle between the distal end of stent and the centerline of the patient’s body was measured by AutoCAD 2014, with the resulting values rounded up to the nearest whole number

## Discussion

Distal migration of biliary plastic stents has been reported in 3–6% of cases [11–13], which is comparable to our result. Previous studies [14, 15] indicated that the majority of distal migrated plastic stents pass spontaneously or can be retrieved endoscopically, and the frequency of duodenal perforation caused by distal migrated stents was less than 1%. A similar frequency of duodenal perforation was also noted in the present study.

Prior studies [12, 13] have suggested that BBS, long stent and proximal stricture are risk factors of distal migration. Similarly, we found that the frequency of distal migration was significantly higher in BBS when compared with MBS. As the stricture in benign diseases is not as tight as that in malignant diseases, the incidence of distal migration is understandably higher in BBS [12, 13]. However, according to multivariable logistic regression analysis, the length of stent and location of stricture did not influence the frequency of distal migration in this study. As is well known, long stents are usually used in proximal strictures; thus, it is unclear whether distal migration is related to stent length or stricture location [13]. Kawaguchi et al. [18] have reported that placement of multiple stents and breaking of stents may result in stent migration. Multiple stents tighten the stricture, thus preventing stent migration. In our center, we usually insert a single plastic stent in patients with BBS first, and then the endoscopist determines if multiple stent placement is required according to the remission degree of stricture after the procedure. In the present study, only patients who underwent placement of a single stent were included; thus, it is difficult to assess this hypothesis. Moreover, stent design and type may also affect the risk of migration. Placement of straight plastic stent with side flaps could reduce the risk of migration [19], so we used such stents in patients. Placement of self-expanding metal stent is recommended for palliative drainage of MBS [4], but many patients in our center prefer to choose plastic stents over the metal stents due to the costs, especially those with a life expectancy of shorter than 6 months. Therefore, plastic stents were used for palliation of MBS in the present study.

In addition to those risk factors, our results revealed that the length of the stent above the proximal end of the stricture played a role in distal migration. The reason we analyzed the role of the length of the stent above the proximal end of the stricture was that this phenomenon was noted in a patient with repeated duodenal injury (patient 5, Table 2). Although there was no established standard for the length of the stent above the proximal end of the stricture, 1–2 cm was regarded as the general choice [20]. If it is too long (> 2 cm, as we presented), the stent may be pushed down and migrate distally after placement, leading to higher risk of duodenal injury. Because the length of the stent outside of the papilla is easy to adjust, adequate assessment of stricture location and selection of appropriate stent length are essential to adjust the length of the stent above the proximal end of the stricture, reducing the possibility of distal migration and its related duodenal injury. Many methods have been suggested for determining the optimal length of the stent [21].

The rate of duodenal injury in cases with distal migration was relatively high in the present study (up to 36%). However, only limited case reports reported distal migration-induced duodenal perforation thus far [6, 7, 17, 22–24]; hence, the risk factors of duodenal injury due to distal migrated stents remain uncertain. Sanchez-Tembleque et al. [17] suggested that the inflexibility of the stent may influence migration and related duodenal injury. Proximal adhesion of the stent to the tumor may increase the intensity of trauma caused by the distal intra-duodenal segment, preventing the stent from adapting to intestinal peristalsis. Adjustment of the length of the stent in relation to the proximal end of the stricture could prevent duodenal perforation. Thus, improper stent length may be a risk factor for injury. However, in our study, the stent length, the length of the stent above the proximal end of the stricture, and the length of the stent outside of the papilla failed to reach statistical significance due to the limited sample size. Instead, we found that duodenal injury occurred when the angle between the distal end of the stent and the centerline of the patient’s body was large (41°), but no injury was noted when the angle was small (20°) in the same patient who underwent repeated placements of biliary stent (patient 7, Table 2; Fig. 1). Further analysis confirmed this finding. We noted that an angle ≥ 30° (the median of angle of patients with distal migration was about 30°) was significantly relevant to duodenal injury. The reasons for the inconsistent angles of the two stent placements in the same patient are unclear. Considering the inability to adjust the angle during the procedure, we suggest that close follow-ups are needed for patients with larger angle to detect duodenal injury, especially when non-specific symptoms like fever and abdominal pain or distension are recorded. Distal migration-induced duodenal injury can present as an early or a delayed complication [6, 23, 25]. In our study, we found that the frequency of distal migration and its related duodenal injury were significantly higher in cases with stent retention < 3 months. One possible explanation is that distal migration and duodenal injury in cases with high risk factors occurred in the early days; thus, the patients were referred to hospital in a short time.

There were several limitations in the present study, including its retrospective nature, single-center design, and small number of cases with available data. Although the sample size is relatively small, the similarity between our results and those previously published support the validity of present data. Further multicenter study with a large sample is needed to verify our results and find more risk factors.

In conclusion, we preliminarily discussed the risk factors of biliary stent distal migration and its related duodenal injury in this study. The risk factors of distal migration are BBS and the length of the stent above the proximal end of the stricture (> 2 cm). Distal migration and related duodenal injury are more likely to present during the early period after biliary stenting. The angle between the distal end of the stent and the centerline of the patient’s body was significantly large in cases with distal migration-induced duodenal injury. In clinical practice, we suggest that endoscopists should select the optimal stent length to achieve an appropriate length of stent above the proximal end of the stricture. Patients with larger angle (≥ 30°) should be monitored closely after stent insertion. Further multicenter studies with large samples are required to identify more risk factors.
